# “Struggling with daily life and enduring pain”: a qualitative study of the experiences of pregnant women living with pelvic girdle pain

**DOI:** 10.1186/1471-2393-13-111

**Published:** 2013-05-13

**Authors:** Margareta Persson, Anna Winkvist, Lars Dahlgren, Ingrid Mogren

**Affiliations:** 1 Dalarna University, School of Health and Social studies, Falun, SE-791 88, Sweden; 2 Sahlgrenska Academy, Dept. of internal medicine and clinical nutrition, University of Gothenburg, Göteborg, Sweden; 3 Department of sociology, Umeå University, Umeå, Sweden; 4 Department of Clinical Science, Obstetrics and gynecology, Umeå University, Umeå, Sweden

**Keywords:** Pelvic girdle pain, Experiences, Pregnancy, Qualitative

## Abstract

**Background:**

Few studies have investigated the experiences of living with pelvic girdle pain (PGP) and its impact on pregnant women’s lives. To address this gap in knowledge, this study investigates the experiences of women living with PGP during pregnancy.

**Methods:**

A purposive sample, of nine pregnant women with diagnosed PGP, were interviewed about their experiences. Interviews were recorded, transcribed to text and analysed using a Grounded Theory approach.

**Results:**

The core category that evolved from the analysis of experiences of living with PGP in pregnancy was “*struggling with daily life and enduring pain*”. Three properties addressing the actions caused by PGP were identified: *i) grasping the incomprehensible; ii) balancing support and dependence and iii) managing the losses.* These experiences expressed by the informants constitute a basis for the consequences of PGP: *iv) enduring pain; v) being a burden; vi) calculating the risks* and the experiences of the informants as *vii) abdicating as a mother.* Finally, the informants’ experiences of the consequences regarding the current pregnancy and any potential future pregnancies is presented in *viii) paying the price and reconsidering the future.* A conceptual model of the actions and consequences experienced by the pregnant informants living with PGP is presented.

**Conclusions:**

PGP during pregnancy greatly affects the informant’s experiences of her pregnancy, her roles in relationships, and her social context. For informants with young children, PGP negatively affects the role of being a mother, a situation that further strains the experience. As the constant pain disturbs most aspects of the lives of the informants, improvements in the treatment of PGP is of importance as to increase the quality of life. This pregnancy-related condition is prevalent and must be considered a major public health concern during pregnancy.

## Background

Pelvic girdle pain (PGP) during pregnancy is a prevalent condition affecting approximately half of all pregnant women [1,2] and for 25 to 30% of pregnant women the condition becomes severe [1-3]. PGP’s aetiology is still unknown and the underlying mechanisms have not been fully elucidated although mechanical, traumatic, hormonal, metabolic, and degenerative factors have been proposed [1,2]. Women who have previously experienced PGP during pregnancy experience a relapse during a subsequent pregnancy in 85 to 95% of cases [3,4], making this pregnancy-related complication a significant condition during pregnancy. Also, a significant number of women suffer from PGP after pregnancy [1,2,5] European guidelines for diagnosis and treatment of the condition are published in 2008. However, despite the condition affecting a large number of pregnancies, inconsistencies regarding definition as well as treatment of PGP still prevail [6].

Previously, most studies have used quantitative methodology to investigate the physical, psychological and socioeconomical implications PGP have during and after pregnancy. For example the physical implications such as: prevalence [3,7], risk factors [3,8], impact of function [7], the significance of previous physical activity [9,10], BMI [11,12], level of pain, and hyper-mobility [12] have been studied. The restrictions of physical activity have been described, where women with lumbopelvic pain in pregnancy report decreased physical ability both in pregnancy as well as after childbirth [13] and 16% of women with self-reported PGP use crutches during pregnancy [7]. The psychological impact of PGP on perceived health, sexual life [5,14] and quality of life [15] is explored as well as the prevalence of sick leave due to PGP [14,16]. Furthermore, exploring the variation of pain catastrophizing (i.e. exaggerated negative orientation toward noxious stimuli [17]) during and after pregnancy, 10.3% report catastrophizing at two occasions during pregnancy and also six months postpartum and for 31.8% of participants the reported catastrophizing varied over time. Women who had reported catastrophizing at least on one occasion during and after pregnancy reported more problems related to lumbopelvic pain and decreased physical ability postpartum [18]. In addition, the experiences of how midwives perceive and experience clients with PGP have been explored using qualitative methods [19]. Because PGP is considered a common clinical problem, midwives have developed a strategy for supporting women affected by PGP; however, mistrust between a midwife and a pregnant woman might occur if vague symptoms are reported [19]. An Australian survey report that 71% of the pregnant women with self-reported lumbopelvic pain have told the health care professionals about their pain, however only 25% receive any type of treatment for their condition. Additionally, some participants express that their condition is normalized by the health care professionals [20]. The pregnant women’s experiences are sparsely investigated using qualitative methodology; existing studies are using written material regarding experiences of treatment programme [21] and discussions in internet forums [22]. As previously shown, PGP has significant implications [2]; however, few studies have explored the experiences of women affected by PGP. This study investigates the experiences of women living with PGP in pregnancy.

## Methods

This interview study used consecutive purposive sampling. A Grounded Theory (GT) approach was applied to elucidate how pregnant women experience PGP. GT applies an abductive approach to generate a theoretical explanation of the phenomena or process of interest; in this case, GT was used to detail the experiences of these women [23]. In GT, the starting point is empirically grounded and may be regarded as naturalistic (ie. reflects people’s ways of receiving and structuring knowledge. Therefore, single experiences may be transformed to broader contexts). The concept of “knowledge of the first and second order” may be applied to understand the experiences of living with PGP. Knowledge of the first order implies common sense knowledge applied to the life world. The knowledge of the second order is the scientific knowledge achieved in a systematic manner and is based on the knowledge of the first order [24]. The study was approved by the Regional Ethical Review Board, University of Umeå, Sweden (Case no 01–249).

### Procedure and informants

Inclusion criteria for the study were pregnant women with verified PGP who were sufficiently fluent in Swedish to be interviewed. At the time of data collection, all women with severe back pain during pregnancy, were referred to the hospital based out-patient clinic for additional examination, diagnosis, and treatment. Prior to the out-patient clinic visit, the pregnant women had been counselled by physicians and/or physiotherapists at the local health care centre; however the treatment available at the local health care centre was not sufficient to ease the problems of these women. One of the authors (IM) worked as an obstetrician on a regular basis at the out-patient clinic and met these women due to her clinical task managing pregnant women with PGP. The examinations used for diagnostics were a detailed medical history, pain palpation tests and pain provocation tests. All women fulfilling the criteria for PGP were offered acupuncture treatment as a complement to any previous treatment. Eligible women were informed of the study after a counselling visit where the diagnosis of PGP was confirmed. If the woman wanted to participate in the study, the medical doctor (IM) did not continue a caregiver-patient relationship after the initial counselling. In such case, the medical responsibility was referred to another obstetrician. Each informant gave her verbal consent after receiving oral and printed information about the aims of the study. In addition, they were informed orally and in writing that participation was voluntary and that they could withdraw from the study at any stage.

Informants were recruited over two time periods. Initially, first-time mothers were recruited in 2002 and 2003 and after initial analysis of the interviews, additional recruitment of pregnant parous women with previous experiences of PGP were performed in 2004 in order to further elucidate the experiences and consequences of PGP in subsequent pregnancies.

Nine informants, aged 27 to 33 years, were purposively recruited for the interviews: four women expecting their first child, four women their second child and one woman expected her third child. All informants were married or cohabiting with their partner and were on full time sick leave from work or studies at the time of the interview. Furthermore, at the time of the interview all informants had experiences of various treatments, for example: analgesics, stability belts, physiotherapy and acupuncture. The interviews were performed in the last trimester of pregnancy (33 – 41 gestational weeks) so as to capture as much information as possible of the experiences of PGP. For more detailed information on informants, see Table 1.

**Table 1:** Characteristics of informants

**Informant No**	**Maternal age at time of interview**	**Profession**	**Educational level**	**Gestational age at time of interview**	**No of children at time of interview**	**Age of previous children at time of interview**
1	27	Teacher	University	36	0	-
2	28	Pre school teacher	University	33	0	-
3	32	Sales clerk	High school	35	0	-
4	33	Civil servant official	University	39	0	-
5	27	Shop assistant	High school	35	1	20 months
6	30	Shop assistant	High school	36	2	4 and 2 yrs
7	32	Teacher	University	41	1	3 years
8	29	Student (maternity leave)	University	35	1	10 months
9	29	Nurse assistant	High school	36	1	16 months

Before the interviews started, the research team prepared a thematic guide that included themes and guidelines for follow-up questions. The main topics of the interview guide addressed experiences of PGP in the current pregnancy including reactions from family, surrounding society, health care professionals and perceived consequences of daily living. Also, reflections and experiences regarding PGP in general were included. The informants were also encouraged to add their own views on the themes and include any aspects and experiences of PGP the interviewer did not address. As the focus of this study was to explore the experiences of living with PGP qualitatively, no additional information of treatments, measurements of pain or effect of received treatments were collected. A pilot interview was performed before the first data collection started to test the thematic guide; the pilot interview results produced a few minor changes to the guide. Prior to the interviews of the parous women, questions regarding aspects of being a mother with PGP and experiences of recurrent PGP were included.

The interviews were performed in a place chosen by the informant; in most cases, they chose to be interviewed in their home. Five interviews were performed by IM and four by another member of the team (AW). All interviews were tape-recorded and lasted for 30 to 90 minutes, mostly 45 to 60 minutes. All interviews contained vivid descriptions of the experiences of PGP during pregnancy. One informant shared one week’s notes from her diary as she thought it would give additional information about the impact PGP had on her situation. This written dairy material was used as a complement to the information provided in that interview. The text from the dairy was coded and analysed in the same way as the transcribed recording of the interview of this informant. After each interview, information from previous interviews helped to improve the next one, and step-by-step, the level of redundancy was reached and the data collection was finalized. After the eighth interview, no substantial new information of the experiences of living with PGP was identified. However, another interview was performed as to confirm that the level of redundancy was obtained. All interviews were transcribed verbatim before the final analysis. The transcribed material comprised 206 pages of text and an additional 12 pages of diary notes.

### Analysis

Our analysis of data followed the Grounded Theory design [25]. By using an abductive approach, closeness to data as well as distancing the data helps to deepen the findings. The first step was the open coding of the transcribed text to create codes in close relation to the text. These codes aimed at discovering ideas about how to generate more abstract concepts and theories from the concrete experiences of the informants and add information to the growing model. During the process of coding and constant comparison, clusters of codes emerged. These groups of similar codes formed categories. Each category comprises new concepts of the experiences described by the codes. As the analysis proceeded, the core category was developed. The core category represents the most prominent properties that can be traced through the most data. The process of analysis proceeded further in search of additional codes and properties to construct an image of how living with PGP during pregnancy was experienced. The theoretical coding started after the identification of the main body of the categories with the aim to link the categories, develop an explanatory model, and explore links to existing theories [23-25].

## Results

The core category that evolved from the analysis of experiences of living with PGP in pregnancy was “struggling with daily life and enduring pain”. Three properties addressing the actions caused by PGP were identified: *i) grasping the incomprehensible; ii) balancing support and dependence* and *iii) managing the losses.* These experiences expressed by the informants constitute a basis for the consequences of PGP: *iv) enduring pain; v) being a burden; vi) calculating the risks* and the experiences of the mothers as *vii) abdicating as a mother.* Finally, the informants’ experiences of the consequences regarding the current pregnancy and any potential future pregnancies is presented in *viii) paying the price and reconsidering the future.* The presentation and discussion will be structured as follows: initially an overview of the conceptual model will be presented, followed by a description of each property illustrated by quotations.

**Figure 1:**
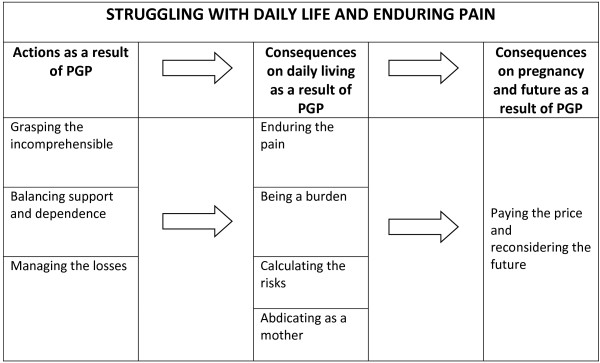
A conceptual model of the actions and consequences caused by PGP as experienced by pregnant informants living with PGP.

Figure 1 presents a conceptual model of the actions and consequences of PGP experienced by the pregnant informants living with PGP. The experience may be regarded as a process of actions and consequences on the management of daily life as well as of the self-image. The perceived incapacity alienated the informants; they could no longer fulfil the roles and expectations of being partners, colleagues and mothers. The strains of living with constant pain, the feelings of incapacity and loss of function due to bodily failure, forced the informants to reconsider their previous ideas of current and future pregnancies.

### Grasping the incomprehensible

Most first-time informants described how they had not known much about PGP, although the condition was not totally unknown. When the first symptoms of PGP occurred, they were unprepared and had problems understanding the situation:

At around the gestational age of 20 weeks I experienced great pain, which was located at the trimmings of the briefs and at the groin. I had no idea of what it was. I changed briefs and bought lots of different kinds – long legs, short legs, cotton or microfiber and without seams, but nothing helped . . . so I have a lot of briefs.

Parous informants with previous experience of PGP expressed that they were not prepared for the deterioration of the condition and did not expect the pain to appear so early in the new pregnancy. Most of the parous informants also noted that the pain was much worse than they could remember from their previous pregnancy.

### Balancing support and dependence

All informants emphasized the importance of receiving psychological and practical support from their partner and others in the close family. Especially for the informants with young children, this support was absolutely essential; to receive help, mutual understanding and cooperation were important aspects to make daily living function. For all informants, household tasks – e.g., babysitting, cooking, cleaning, and rides to the midwife or doctor appointments – were redistributed to partners, parents and other relatives. Sometimes, this position of increasing dependence resulted in stress both in the relationships with their partner and with other members of the immediate family:

I have a lower tolerance for mess and dirt in the household than he has. He doesn’t think this is important and then I get angry and start cleaning and tidying up. Then I get more pain and become bad tempered. He should understand; I should not have to tell him. It’s remarkable, but he doesn’t understand.

I have been so mad at him as he’s not helping me out at home; in some way it’s expected that I should manage.

### Managing the losses

The pain contributed to changes, sometimes disturbing and agonising changes, in many aspects of the informant’s life. For example, the informant’s sexual life was affected; informants indicated that their sexual life during pregnancy was characterized by a decrease in sexual activity and desire. Some described a dysfunctional sexual life characterized by guilt and sexual frustration – both their own as well as their partners’:

I had heard stories of improved sexual life during pregnancy and I had really looked forward to that. And then nothing like that occurs, and I feel it is my fault since it depends on me.

All informants had strong professional identities and their work played a significant role in their lives. They felt their sick role as a deviation, a temporary state that had previously been relatively unknown. Initially, they struggled on at work despite comments and feelings of incapacity. One women expressed:

“*It feels like my colleagues have to work harder as I’m incapable and then one of my colleague, who has no family nor has been pregnant, has huge problems to understand what this is like… she once said to me: I’ve never met someone panting as much as you do”*

Some informants were able to adjust their work tasks and have a partial sick leave to start with but eventually, all informants were on full-time sick leave. Although they had problems accepting this new status of incapable to work, they were often relieved as the sick role allowed them the opportunity to rest. In addition, the hope and expectation of enjoying the pregnancy were diminished.

“It has been nice to pregnant from a psychological aspect, but to experience that the body fails is so sad”.

Furthermore, the pain was physically exhausting although some experienced that their mental capacity remained unaffected. The pain influenced almost all daily activities so their mood and satisfaction were negatively affected. Some experienced vulnerability and losing the sense of coherence and togetherness that comes with being a part of a team at work. The informants described feelings such as guilt, shame, frustration, and resignation caused by their condition and inability to fulfil the expectations and obligations of being a wife/partner as well as a colleague at work. These aspects may also be described as social and personal losses; the sick roll diminished their status in the society and put strains on their relationship.

I feel incredibly alert mentally although my body hurts. I have sometimes felt that they do not believe that I’m in pain.

I’m meeting so much people daily at work, and then just ending it suddenly. I would go crazy, I am going crazy!

You are nobody when you are sick.

### Enduring the pain

Some informants perceived the start of the pain as sudden whereas others found it developing progressively. A few informants with previous children experienced the first lingering symptoms even before the classic symptoms of pregnancy. All informants described how increasing pain problems resulted in increasing restrictions on daily activities, both within their professional environment and the household. For some, the level of pain increased during pregnancy until it reached a level of almost constant pain:

And when I went to bed, I felt it was creeping all over my body and I had an enormous pain, which was inconceivable.

I thought I would break into two parts.

The pain was experienced as exhausting and gnawing, resulting in an increased need for rest. However, the constant pain affected the quality of sleep and ability to rest and recover.

“*I wake up every time I turn over in bed at night”.*

The constant pain was perceived as invisible to others, a perception that was the source of further anxiety and frustration as some informants experienced scepticism from their surrounding.

“*I don’t think that everyone knows the difference between pelvic pain during pregnancy and just an aching back”.*

### Being a burden

Desire for independence was a common feature of the informants. They described themselves as being competent and qualified in their professional and private lives. In the current situation, they were far from their normal level of productivity and performance. The process of realizing and accepting their limitations was acknowledged as agonizing by some informants. The informants were now dependent on others. This could be described as a major internal conflict in which their self-image was distorted.

My god, pregnancy is not a disease. Everything ought to be as before the pregnancy, sometimes I cry myself to sleep.

It’s devastating to be a married 27 year old with a family of my own and be dependent of my parents to make my daily life function. It really gnaws my self-esteem.

### Calculating the risks

All informants used different strategies to minimize the pain symptoms in relation to various daily activities. They described a learning process over time to avoid activities that might provoke more pain. This was very difficult to achieve for the informants with young children, as the children’s needs had to be cared for no matter how much pain they experienced themselves. A few of the informants, for example, described how they taught their toddlers to climb the stairs in the house in a safe manner as to avoid having to carry them. All informants described how it was necessary to plan the movements and activities in advance and all activities were valued in relation to the amount of pain expected.

“One has to assess – How much pain is this worth?”.

### Abdicating as a mother

The informants with young children described how the immobility and the pain contributed to feelings of not being able to be a proper mother. They only managed to be on their own with their children for short periods, which resulted in a fear of not managing upcoming situations, such as not being able to catch the child running off or to lift the child if necessary. Furthermore, they expressed that the pain made them unable to cope with the needs of closeness and attention of the children. These situations made the mothers really sad, as they could not satisfy their children’s needs:

Over and over again, she came and wanted me to play. Pooh, each time it breaks my heart to have to say: Mummy cannot. You have to play on your own or with whoever helps me out this day. She looks just as disappointed each time, but so far she asks me. I dread the day she’ll stop asking me since I am almost never ever able, manage or have the energy to play with her. To not be able to be the mum I want to be is the absolute worst in this situation.

A couple of the informants with young children expressed that they regretted their pregnancy, a situation that resulted in considerable psychological strain. A baby was planned and longed for, but the journey towards the birth was so stressful that they wondered how they would manage.

“*There are moments when I find myself wishing that I should not have become pregnant again”.*

### Paying the price and reconsidering the future

Overall, the wellbeing of the foetus was the primary concern and the informants’ own wellbeing was of secondary importance. The loss of function, however, decreased the joy of pregnancy although they were all assured that the baby’s wellbeing was not affected by the condition. The informants just endured and looked forward to the birth of the baby. Hopefully, the pain would vanish instantly after birth and life would go back to normal again. However, most informants feared that the condition might be chronic. Moreover, even if they recovered, the fear of an even more aggravated situation in a new pregnancy made them reconsider their dreams of having a larger family:

Never ever, once more. In that case, my husband would have to have a child with another woman (laughs). It’s not going to happen.

As I feel right now, never more I will torture my body like this.

## Discussion

### Methodological considerations

Nine pregnant women with diagnosed PGP were recruited to the study over two periods. The first period focused on the experiences of first-time mothers and the second focused on deepening and enhancing the experience by focusing on parous women with previous experiences of PGP. The data collection continued until the level of redundancy was achieved, i.e., no substantial new information was discovered in relation to previous findings during data collection and analyses. The informants were recruited by one of the authors (IM) who worked as an obstetrician in the out-patient clinic. The invitation to participate in the study was put forward after the counselling was completed. IM did not give any further medical counselling after their first visit. This was to assure that the informants were not in a dependent relationship with the author.

Two of the authors (MP and IM) have vast experience helping pregnant women deal with PGP during and after pregnancy. Generally, extensive pre-understanding may be disadvantageous during data collection and analyses in qualitative research. However, the researchers were aware of this situation early on and the constant involvement of the two co-authors without this experience counterbalanced this pre-understanding and made it possible to explore and discuss the data in an balanced manner.

As all informants were referred to out-patient clinic counselling due to their situation, it is likely that they comprise a selected group of women with more advanced symptoms than most others in the population. However, few symptoms described were unfamiliar to the experienced authors or not described in the literature previously; hence the general medical picture of the informants was consistent with the pre-understanding achieved when counselling women with PGP on a regular basis. Furthermore, all informants were examined by an experienced obstetrician, ensuring that the pain problems these women were experiencing were related to PGP and no other back or pelvic problems. Results from a small sample of informants cannot be generalised; however, the experiences and impacts on daily living reported by the informants in this interview study may be recognised by other women suffering from PGP and living in similar contexts.

There is always a risk of an overrepresentation of talkative individuals when recruiting for interview studies. The variation in lengths of interviews might indicate that a variety of informants were represented. Furthermore, socioeconomic status depending on profession, education and employment conditions may influence the experiences of the women. Some of the informants were able to work part-time for several weeks before full sick leave as there were possibilities to adjust work tasks as well as working positions, while others had no such opportunities which resulted in sick leave as the work could not be adjusted to the individual. The various backgrounds, educational levels as well as professions representing a range of common professions among women contributed to the rich material. As to validate the findings, a thick description is provided that summarizes the codes, and quotations have been used to illustrate the properties.

### Major findings

The findings of this study elucidate the stress and feeling of incapacity in many aspects of life women with PGP may experience during pregnancy. The core category “Struggling with daily life and enduring pain” embraced the actions and consequences the informants had to manage. The perceived incapacity had impact on many aspects of life resulting in a daily struggle to obtain an individual balance of adjusted activity and rest without increasing the pain. In Sweden, the societal opinion of pregnancy is that it is a normal condition implicating that a woman with a normal pregnancy should be able to continue her life as usual during pregnancy. The view of pregnancy as a normal condition where pregnancy-related problems not necessarily should be a reason for sick leave is widely spread, for example stated by the information available at the web site of the Swedish Social Insurance Agency [26]. However, in various Swedish studies and reports, the prevalence of sick leave in pregnancy is reported to 39% - 52% [15,27,28]. A considerable part of these women are on sick leave due to back or pelvic girdle pain [27,29]; hence a substantial number of pregnant women are affected each year.

Previously, quality of life in pregnant women with back pain has been studied using quantitative methods. Findings show that pregnant women with back pain rate their total health – related quality of life significantly lower than pregnant women without back pain in late pregnancy. The significant differences are also present regarding some of the subscales (sleep, energy, pain and physical mobility). Additionally, women with back pain report increased negative affection on occupation, ability to perform jobs around the house, social life and hobbies than women without back pain in late pregnancy [15]. Further, the association of poorer sleep and back pain in pregnant women is described. These quantitative findings of poorer health-related quality of life are similar to the results of this interview study. Our informants expressed their experiences of bodily failure leading to sick leave and dependence of others to manage their daily life. Also, the informants expressed how the pain disturbed their ability to sleep and recover. Other studies have shown that sleep difficulties in pregnancy may be related to increased levels of depressive symptoms later in pregnancy [30], and in a population-based study of insomnia and depression in late pregnancy, the presence of PGP and lower back pain were significantly related to insomnia, but not associated with depressive symptoms [31]. Furthermore, emotional distress during pregnancy in women with PGP is associated with persisting pelvic girdle syndrome postpartum in a Norwegian study. The more severe pain problems during pregnancy, the less recovery rate six months postpartum. Furthermore, reporting emotional distress at both gestational weeks 17 and 30 show associations with persistent pelvic pain syndrome [32]. The impact of pain and decreased ability to fulfil the expectations from family and colleagues expressed by the informants may contribute to an increased vulnerability and risk of developing depressive symptoms. Also, as a number of women experience catastrophizing during and after pregnancy [18], this could also contribute to the experiences expressed. An overall assessment of sufficient pain management should be considered in all clinical consultations of pregnant women with PGP as the findings from this and other studies indicate that the experience of persistent pain contributes to sleep problems, poorer quality of life and may contribute to increase depressive symptoms.

The literature on the experience of living with PGP and its impact on daily life are limited. To our knowledge, there are no previous qualitative publications that investigate the impact of PGP on daily life experienced by pregnant women. A qualitative Norwegian study of women’s contributions to Internet-based discussion forums show that the condition of PGP is perceived as unpredictable and disabling [22]. The findings explore to what extent one should endure; the women participating in the forums advise each other to rest and be careful so as not to make the situation worse. Using wheel chairs or crutches was seen as the worst case scenario and women fear that their condition would not improve after childbirth [22]. Although it is likely that not all the women participating in the Internet forums suffer from PGP, the findings correspond to the experiences of our informants with a diagnosis of PGP. Our core category “Struggling with daily life and enduring the pain” may be applied on these findings as well. The Internet-based discussions address the unpredictability and disability related to PGP; subjects very similar and significant to the experiences expressed by our informants.

The experiences of women living with chronic bodily pain, such as fibromyalgia, have been studied and these findings show some similarities with women with PGP in pregnancy. Söderberg et al. (1999) performed an interview study with 14 women living with fibromyalgia. The women revealed an experience understood as three connected themes: loss of freedom, threat to integrity, and a struggle to achieve relief and understanding [33]. Furthermore, the experience of fibromyalgia pain was expressed as a double burden: coping pain that can be unpredictable and destructive as well as managing doubts from others, as the condition causing the pain is invisible. All informants strived to normalize their daily life by finding ways to distract the pain by doing joyful tasks experienced as worthwhile and reconciling living with the constant pain by finding other ways of dealing with pain [34]. These findings of living with chronic pain conditions have many similarities with the findings of our study despite the differences in conditions and circumstances.

Joachim and Acorn (2000) present a preliminary framework of the stigma of visible and invisible chronic conditions, which indicates that being different from the population in general might lead to stigmatization, so the individual with a chronic condition chooses strategies to either reveal or hide the condition [35]. Pregnancy comes with many expectations, norms, and values set by society; for instance, the previously mentioned statement that pregnancy is a normal condition [26]. Applying this framework to the situation of pregnant women with PGP, the haunted women either choose a strategy to reveal their condition (i.e., the invisible pain) and hence risk additional stigma, such as doubt and distrust from others, or try to hide the condition for as long as possible so as to pass as normal, hence fulfilling the societal expectations of a pregnant woman. Most of our informants had to reveal their condition as it deteriorated (poor walking, increasing pain, etc.). Despite obvious symptoms, many experienced that some people questioned the extent of their pain and condition. We have previously described that midwives working at local health centres usually provide the initial support and counsel pregnant women with PGP. However, when uncharacteristic or vague symptoms of PGP are present, the midwives recognize that there may be doubts or distrust towards the accuracy of the condition of the pregnant woman [19].

The findings of this study indicate a need for knowledge that will help improve the care provided for these women and also improve the understanding of PGP and its impact on pregnant women’s daily lives. This area needs to be investigated further using the views and experiences of pregnant women. Additionally, as the condition have an impact on the relationship with the partner of the affected women, investigations of how the condition of PGP is experienced from their partners’ view may further contribute to the knowledge of this field.

## Conclusion

PGP during pregnancy greatly affects the woman’s experiences of her pregnancy, her roles in relationships, and her social context. For women with young children, PGP negatively affects the role of being a mother, a situation that further strains the experience. As the pain disturbs most aspects of the lives of these women, improvements in the treatment of PGP is of importance as to increase the quality of life in these women. This pregnancy-related condition is prevalent and must be considered a major public health concern during pregnancy.

## Abbreviations

PGP: Pelvic girdle pain; GT: Grounded theory.

## Competing interests

The authors declare that they have no competing interests.

## Authors’ contributions

AW, LD and IM contributed to the design of the study. AW and IM performed the interviews. MP analysed the collected data and developed the draft of the manuscript. All authors provided critical feedback to the interpretation of data and the development of the final manuscript. All authors have read and approved of the final manuscript

## Authors’ information

The first author, MP (woman), is a qualified midwife with experience in providing antenatal health care. One co-investigator, IM (woman), is an obstetrician active in reproductive health research. The other two co-investigators, AW (woman) and LD (man), are both active in public health research. AW is a nutritionist with a research focus on reproductive health. LD is a medical sociologist with a research focus on the sociology of emotions.

## Pre-publication history

The pre-publication history for this paper can be accessed here:

http://www.biomedcentral.com/1471-2393/13/111/prepub
